# *JAK2* Mutations Are Rare and Diverse in Myelodysplastic Syndromes: Case Series and Review of the Literature

**DOI:** 10.3390/hematolrep15010008

**Published:** 2023-01-18

**Authors:** Melissa Delio, Christine Bryke, Lourdes Mendez, Loren Joseph, Sarmad Jassim

**Affiliations:** 1 Department of Pathology, Beth Israel Deaconess Medical Center, Boston, MA 02215, USA; 2 Division of Hematology/Oncology, Beth Israel Deaconess Medical Center, Boston, MA 02215, USA; 3 Department of Pathology, William Beaumont Hospital, Royal Oak, MI 48073, USA

**Keywords:** *JAK2* mutation, MDS, myelodysplastic syndrome, deletion 7q

## Abstract

Objectives: To investigate and characterize *JAK2* mutations in myelodysplastic syndrome (MDS), we present three cases with diverse *JAK2* mutations and review the literature. Methods: The institutional SoftPath software was used to find MDS cases between January 2020 and April 2022. The cases with a diagnosis of a myelodysplastic/myeloproliferative overlap syndrome including MDS/MPN with ring sideroblasts and thrombocytosis were excluded. The cases with molecular data by next generation sequencing looking for gene aberrations commonly seen in myeloid neoplasms were reviewed for the detection of *JAK2* mutations including variants. A literature review on the identification, characterization, and significance of *JAK2* mutations in MDS was performed. Results: Among 107 cases of the MDS reviewed, a *JAK2* mutation was present in three cases, representing 2.8% of the overall cases. A *JAK2 V617F* mutation was found in one case representing slightly less than 1% of all the MDS cases. In addition, we found *JAK2 R564L* and *JAK2 I670V* point mutation variants to be associated with a myelodysplastic phenotype. Conclusions: *JAK2* mutations in MDS are rare and represent less than 3% of cases. It appears that *JAK2* variant mutations in MDS are diverse and further studies are needed to understand their role in the phenotype and prognosis of the disease.

## 1. Introduction

In this manuscript we attempt to investigate and characterize *JAK2* mutations in myelodysplastic syndrome (MDS). We will begin with a review of the literature followed by the incidence of *JAK2* mutations in MDS patients found at our institution. We will complete the manuscript with a presentation of three different patients with a first-time diagnosis of MDS with diverse *JAK2* mutations.

**Key point:**

- ***JAK2 V617F* mutation is rare in myelodysplastic syndromes and in its presence a myeloproliferative disease needs to be excluded.**
- ***JAK2* mutations are diverse and *JAK2* variant mutations may lead to a myelodysplastic syndrome phenotype.**
- ***JAK2 R564L* and *JAK2 I670V* are reported as *JAK2* mutation variants in association with a myelodysplastic phenotype.**
- **Further studies are recommended to investigate the relationship between and significance of *JAK2* mutations variants and their clinico-morphologic phenotypes.**

*JAK-STAT* signaling plays a major role in cancer evolution including myeloid neoplasms as a result of increased Janus Kinase (JAK)-mediated activation of downstream oncogenic factors [1]. Somatic activating mutations in *JAK2 V617F* located at 9p24 are very common in myeloproliferative neoplasms (MPN) and have been associated with an increased incidence of thrombosis, hemorrhage, and fibrosis [1,2,3]. In the general healthy population, *JAK2 V617F* mutations are rare affecting only 0.1–0.2% and are associated with increased morbidity and mortality [2,4]. In the study by Nielson et al. from a Danish general population cohort, while the majority of *JAK2* positive healthy participants had a low mutation burden below 12%; it has been found that an increased *JAK2 V617F* mutation burden is associated with increased erythrocytes, platelets, and leukocytes [4]. In addition, the same study found an association of the *JAK2 V617F* somatic mutation with age implying an overtime status change [4]. 

The relationship between *JAK2 V617F* mutations and hematopoiesis is complex. In one study, on 43 patients with essential thrombocythemia (ET), it was found that megakaryocytes and bone marrow mononuclear cells exhibit the inhibition of caspase-dependent apoptosis leading to the accumulation of megakaryocytes [5]. In addition, the authors report a higher Bax expression and activation of Cas-3 in *JAK2 V617F*-mutated cases when compared to *JAK2* negative cases [5]. The interaction between different upstream cytokine receptors and the *JAK-STAT* downstream signaling cascade can become dysregulated causing aberrant *JAK* activation. Aberrant *JAK* activation impacts the survival, maintenance, differentiation, and proliferation of hematopoietic cells including erythroids, granulocytes, and megakaryocytes and leads to the variable phenotype of myeloid neoplasms [6]. Different mutations can affect any component of the *JAK-STAT* pathway driving the phenotype leading to different hematological neoplasms [7]. For example, while the *JAK2 V617F* mutation is the most common, particularly in MPN, a gain of function mutation in exon 12 of *JAK2* is characteristic for polycythemia vera (PV) and is not commonly seen in primary myelofibrosis (PMF) or ET [7,8]. Less common *JAK2* variants/mutations located anywhere along the four main domains of the *JAK2* structure have been described and the interpretation of such rare variants continues to be a challenge [8]. 

The phenotype of myeloid neoplasms may depend on the intensity of signaling on specific downstream targets in the *JAK-STAT* signaling pathway, which is related to the number of *JAK2 V617F* copies and the presence of additional gene mutations affecting RNA splicing genes or epigenetic regulators [7]. For example, it is known now in MDS/MPN with ring sideroblasts and thrombocytosis (MDS/MPN-RS-T) that the *JAK2 V617F* mutation drives the thrombocytosis while the *SF3B1* mutation drives the increased ring sideroblasts [7]. Furthermore, phenotypic switching has been associated with additional *JAK2* mutations within hematopoietic cells [8]. In one study, 35 unique *JAK2* variants were reported across different functional domains and specific *JAK2* variants detected in MPNs may predict evolution to acute myeloid leukemia [8]. Additionally, non-canonical *JAK2* and *MPL* mutations have been reported in myelofibrosis and are not limited to triple negative MPN [9].

*JAK2 V617F* mutations are rare in MDS and present in approximately 2–5% of cases [10,11,12,13]. In one study including 53 MDS patients, the *JAK2 V617F* mutation was present in 3 cases. These three cases included one patient with refractory anemia and two patients with refractory anemia with ring sideroblasts and thrombocytosis, identifying a subset of patients with overlap features including proliferative bone marrow, thrombocytosis, and leukocytosis [10]. In another study on 439 patients with MDS, a *JAK2* mutation was found in 13 patients at approximately a 3% frequency [12]. 

*JAK2 V617F* mutations have been reported to occur at a higher frequency in MDS with isolated 5q deletion and cases of MDS/MPN with ring sideroblasts and thrombocytosis [10,11,14,15,16,17]. In one study on 47 cases of MDS with an isolated del(5q), 6 cases (12.7% of total cases) had a concurrent *JAK2 V617F* mutation [14]. Another study that included 97 cases, found that 6 cases (6.2% of total cases) revealed a concurrent mutation [15]. A third study included 88 cases with myelodysplastic syndrome and an isolated del(5q) in which 5 cases (6.4% of total cases) revealed concurrent *JAK2 V617F* mutations [17]. Interestingly, while some studies found that the association did not seem to affect phenotype or prognosis [17]; other studies showed patients exhibited heterogeneous blood cell counts with a trend to higher white cell counts and platelet counts, a hypercellular bone marrow with variable dysplasia, and increased pleomorphic megakaryocytes including del(5q) such as megakaryocytes as well as large hyperlobated megakaryocytes suggesting features of an overlap MDS/MPN disease [14,15]. In one study, the lower mutation burden of *JAK2* in comparison to *SF3B1* mutation led the authors to suggest that most RARS-T evolved from RARS/RCMD-RS with subsequent acquisition of a *JAK2* mutation [13]. 

In addition, some cases of MDS with fibrosis have been associated with a *JAK2 V617F* mutation suggesting that this may be responsible for myelofibrosis in a subset of MDS cases, postulating a possible myeloproliferative biology background [18]. In some studies, *JAK2* was found to be more frequent in the low risk WHO MDS subtype [13]. In a meta-analysis of 150 cases to compare gene mutational profiles across MDS, MDS/MPN, and MPN, the results confirmed the low frequency of *JAK2* mutations in MDS and its more frequent occurrence among myeloproliferative neoplasms and cases with overlap features, particularly MDS/MPN-RS-T [19]. Different studies additionally confirmed the *JAK2 V617F* activating mutation is infrequent in atypical MPN cases and overall MDS patients [20]. In some situations, the findings are challenging and may lead to a diagnostic dilemma for instance within a fibrotic marrow with features indeterminate for MDS or MPN. In these cases, some authors have suggested that *JAK2 V617F* allele evaluation is a useful ancillary test for this discrimination [21]. In other studies, *JAK2 V617F* mutations were found to be unlikely to play a significant role in the pathophysiology of MDS with or without secondary fibrosis and it was suggested that strict criteria should be applied to exclude the more likely diagnosis of MPN in transformation when *JAK2 V617F* is detected in patients with dysplasia and myelofibrosis [22]. 

The prognostic implication of *JAK2 V617F* mutations is unknown and controversial in MDS, variable in MPN, and in MDS/MPN RS-T carries a better prognosis. Some studies attempted to clarify the prognostic significance of such mutations in MDS. In one study on 132 patients with MDS, a *JAK2* mutation was associated with a lower rate of progression to acute myeloid leukemia, a better overall survival, and possibly a favorable prognosis [23]. However, another study that included 1514 patients undergoing allogenic hematopoietic stem cell transplant with MDS revealed that *TP53* mutations, *RAS* pathway mutations, and *JAK2* mutations were associated with a shorter survival [24] While the mechanism of the *JAK2* mutation is unknown, interestingly, the patients with *JAK2* mutations were associated with a higher rate of death without relapse regardless of conditioning intensity suggesting the significance of using *JAK2* inhibitors in this subset of patients [24] (Table 1).

## 2. Materials and Methods

Our institutional SoftPath software was used to find cases with an MDS diagnosis between January 2020 and April 2022. The cases with a diagnosis of MDS/MPN with ring sideroblasts and thrombocytosis (MDS-MPN-RS-T) were excluded and the 2017 WHO criteria were followed strictly. The cases with molecular data by next generation sequencing looking for gene aberrations commonly seen in myeloid neoplasms were reviewed for the detection of *JAK2* mutations including variants. A literature review on the identification, characterization, and significance of *JAK2* mutations in MDS was additionally performed.

## 3. Results

Our search yielded 341 cases with the word myelodysplastic syndrome included in the report. These reports were reviewed comprehensively and 107 cases were found to meet the criteria for a diagnosis of myelodysplastic syndrome and were included in our study. Among these 107 cases of MDS reviewed, a JAK2 mutation was present in 3 cases representing 2.8% of the overall cases. However, a JAK2 V617F point mutation was found in one case representing slightly less than 1% of the overall cases. In addition, we found JAK2 R564L and JAK2 I670V point mutation variants to be associated with the other two cases with a myelodysplastic syndrome phenotype.

### 3.1. Cases Presentation

#### 3.1.1. Case 1

The patient is a 53-year-old male with a 25-year smoking history who presented with progressive moderate anemia from 2019 and intermittent leg pain at night for two years, as well as paresthesia in his feet. He has had an extensive work-up that was unrevealing and an initial bone marrow biopsy at an outside institution with a presumptive diagnosis favoring aplastic anemia. 

**Figure 1:**
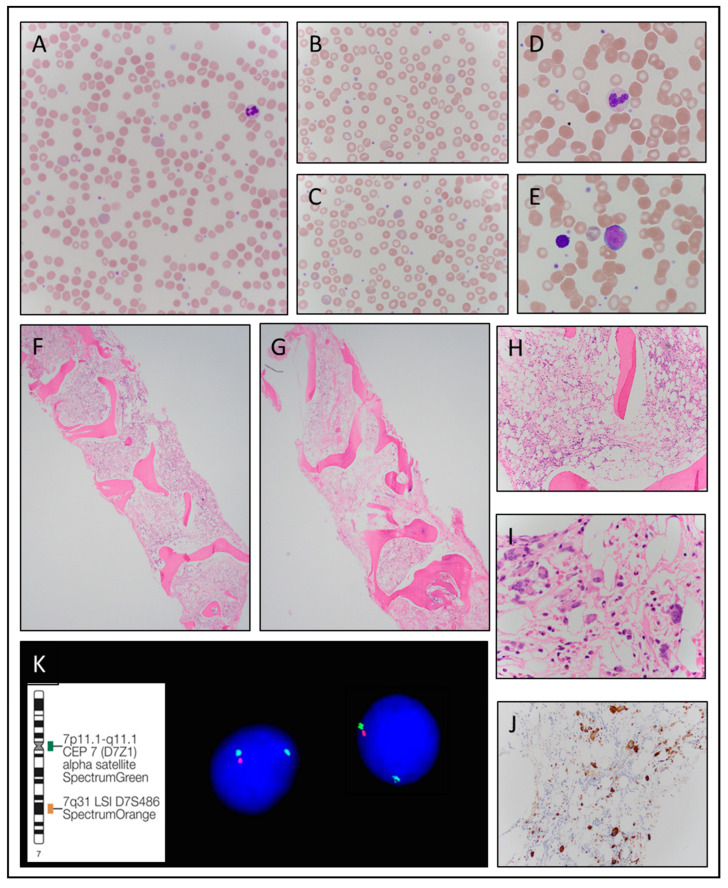
Case 1. (**A**) Peripheral blood smear reveals a dimorphic population. (**B**) Occasional tear drop red blood cells (60× oil). (**C**) Scattered red cells with basophilic stippling (100× oil). (**D**) Occasional neutrophils with Döhle-like bodies (100× oil). (**E**) Occasional circulating blasts (100× oil). (**F**,**G**) Core biopsies with hypocellular for age erythroid dominant trilineage hematopoiesis (2×). (**H**) Hypocellular for age erythroid dominant trilineage hematopoiesis (10×). (**I**) Loose clusters of megakaryocytes including different forms (60× oil). (**J**) CD42b highlights megakaryocytes including small hypolobated forms (40×). (**K**) Fluorescence in situ hybridization (FISH) analysis of interphase peripheral blood cells with abnormal probe signal pattern consistent with deletion of the 7q31 region using the Abbott Molecular D7S522 IL, USA and CEP7 dual color probe set with Spectrum Orange directly labeled D7S522 probe for the 7q31 region and Spectrum Green directly labeled probe for the centromeric region of chromosome 7.

At presentation to our institution, the patient had a normocytic normochromic anemia with a hemoglobin of 9.5 g/dL and a MCV of 89 fl. The review of the peripheral blood smear revealed marked anisopoikilocytosis with a dimorphic population including macrocytes, occasional tear drops, basophilic stippling, and polychromasia (Figure 1A–C). Additionally, his white blood cell, absolute neutrophil, and platelet counts were normal (Table 2). His LDH was normal at 164 IU/L. His spleen was initially within normal limits, however, following imaging 6 months later his spleen was found to be mildly enlarged at 14.9 cm. Minimal lymphadenopathy in the bilateral internal iliac and external iliac lymph node chains were also reported at this time. 

Occasional circulating blasts were identified intermittently on two occasions at 1–2% and rare granulocytes showed abnormal granulation and Döhle-like bodies (Figure 1D,E). The patient underwent several bone marrow biopsies. Unfortunately, the bone marrow aspirates were often inadequate due to difficult aspiration and marked hemodilution rendering them insufficient for definitive cytomorphologic evaluation. The bone marrow core biopsies were markedly hypocellular for age (Figure 1F,G) at approximately 10% overall cellularity with a focally serous atrophic background (on initial biopsy), erythroid dominant hematopoiesis, and scattered megakaryocytes with focal loose clustering (Figure 1H,I). Using CD34 staining, less than 5% scattered blasts were identified and CD42b highlighted slightly increased megakaryocytes with occasional small forms and focal loose clusters (Figure 1J). A Reticulin stain revealed MF-2 to MF-3 of 3 WHO grade fibrosis and the trichrome highlighted focal grade 1–2 collagen fibrosis. 

Concurrent flow cytometry on peripheral blood showed no immunophenotypic features of involvement by lymphoma or acute leukemia and the high sensitivity paroxysmal nocturnal hemoglobinuria (PNH) panel was negative for a PNH clone. Cytogenetics on the peripheral blood repeatedly showed an abnormal karyotype with a derivative chromosome that resulted in 7q- and an extra copy of 1q. Fluorescence in situ hybridization (FISH) analysis was performed using the Abbott Molecular D7S522 and CEP7 dual color probe set. The Spectrum Orange directly labeled D7S522 probe for the 7q31 region in combination with the Spectrum Green directly labeled probe for the centromeric region of chromosome 7 detected deletions of the 7q31 region and was repeatedly positive for the deletion of 7q31 in 4% and 14% of the interphase peripheral blood cells examined (Figure 1K). Given the cytogenetic findings, a presumptive initial diagnosis of a hypocellular myelodysplastic syndrome with myelofibrosis not further classified was rendered. Subsequent next generation sequencing looking for myeloid neoplasm associated gene mutations showed a concurrent *JAK2 V617F* mutation with a low allele variance frequency of 8%, causing this case classification to be more challenging. Telomere length measured on peripheral blood using flow cytometry and FISH was within the normal range. An inherited bone marrow failure molecular panel was reported as indeterminate with two heterozygous variants detected in *TET2* and *SAMD9*. 

#### 3.1.2. Case 2

The patient is a 60-year-old female with a past medical history of hypothyroidism, depression, and long-standing relapsing/remitting multiple sclerosis, now in remission, whom presented four years ago with progressive macrocytic anemia of 9–10 years. Of note, the patient does not have a history of previous exposure to chemotherapy or irradiation. 

The patient’s hemoglobin at the time of the initial diagnostic bone marrow biopsy was 9.6 g/dL with an MCV of 110 fl that progressively worsened to 8.4 g/dL on her second diagnostic bone marrow biopsy. There was no evidence of leukocytosis or thrombocytosis at any point over more than 10 years of complete blood cell counts at our institution. The LDH was normal at 178 IU/L and has not shown any increase before presentation or during management. There was no evidence of splenomegaly by examination and the imaging revealed mild stable splenomegaly with stable splenic calcification. 

**Figure 2:**
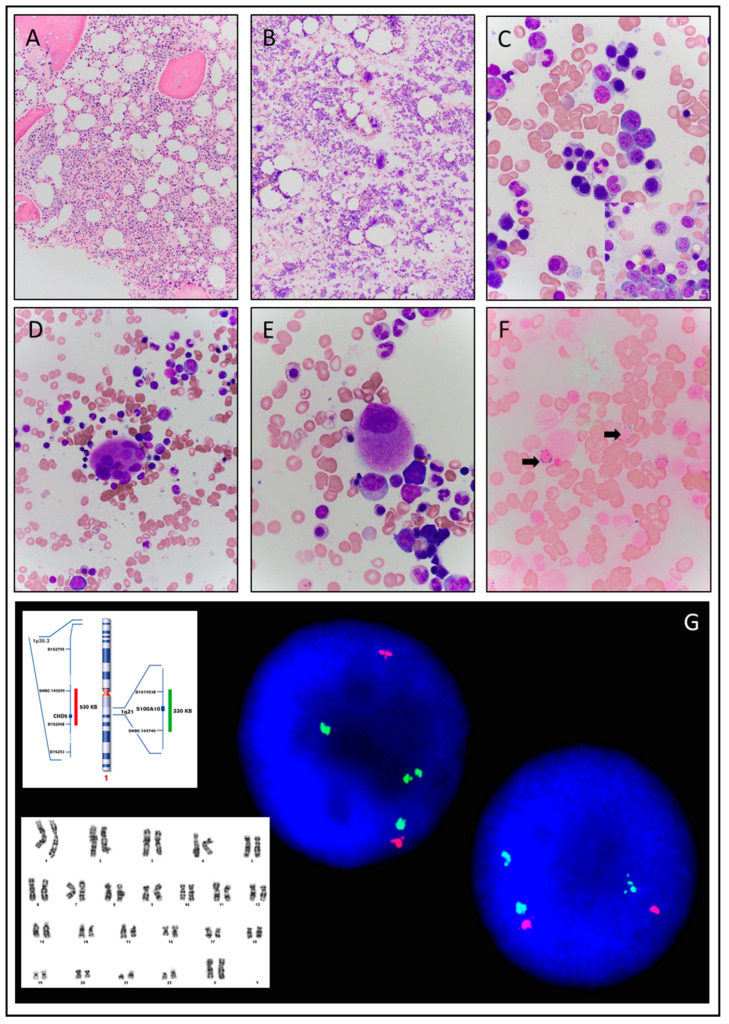
Case 2. (**A**) Bone marrow core biopsy with approximately 50% cellularity (40×). (**B**) Bone marrow aspirate with erythroid dominant trilineage hematopoiesis and slightly pleomorphic megakaryocytes (10×). (**C**) Erythroid dominant hematopoiesis with dysplasia including erythroids with irregular nuclear contours, binucleation, satellite nuclear fragments, megaloblastic maturation, and nuclear blebs (100×). (**D**) Hyperlobated megakaryocyte with disjointed nuclear lobe (100×). (**E**) Hypolobated megakaryocyte (100×). (**F**) Iron stain revealing markedly increased ring sideroblasts (black arrows) (100×). (**G**) Abnormal female karyotype 46,XX,dup(1)(q21q32) [11]/46,XX [9] with duplication of 1q and fluorescence in situ hybridization (FISH) analysis with the Kreatech 1p36 and 1q21 dual color probe set shows a gain of the long arm of chromosome 1 in the interphase bone marrow cells examined.

There were no circulating blasts reported and the bone marrow aspirate and core biopsy showed erythroid dominant trilineage hematopoiesis with variable trilineage dyspoiesis (Figure 2A,B). Dysplasia was most prominent within the erythroids that demonstrated nuclear irregularities, nuclear budding, binucleation, satellite nuclear fragments, and megaloblastic maturation with nucleo-cytoplasmic maturation asynchrony (Figure 2C). Granulocytes revealed complete maturation with occasional hypogranular mature forms and granulocytes with polarization of granules. The megakaryocytes were increased in number and exhibited a slightly pleomorphic morphology including large hyperlobated forms with disjointed nuclear lobes and occasional hypolobated forms (Figure 2D,E). The blasts represented approximately 1% of the overall differential counts. An iron stain performed on the bone marrow aspirate revealed numerous ring sideroblasts at more than 40% of nucleated red cells (Figure 2F). The bone marrow core biopsy was cellular at approximately 50–60% overall cellularity with an erythroid-dominant trilineage hematopoiesis (Figure 2A). Given these findings, the patient was rendered a diagnosis of myelodysplastic syndrome with ring sideroblasts and multilineage dysplasia (MDS-RS-MLD). 

Concurrent flow cytometry on the bone marrow aspirate detected approximately 2% blasts and no immunophenotypic evidence of leukemia or lymphoma. Conventional cytogenetics yielded an abnormal karyotype with a duplication of the long arm of chromosome 1 in eleven cells of the twenty mitotic cells evaluated (Figure 2G). An MDS FISH panel was negative with no evidence of interphase bone marrow cells with the common cytogenetic abnormalities observed in myelodysplastic syndrome that include deletion 5q31, deletion 7q31, monosomy 7, trisomy 8, and deletion 20q12. However, fluorescence in situ hybridization (FISH) analysis with the Kreatech 1p36 and 1q21 dual color probe set showed a gain of the long arm of chromosome 1 in 27% of the interphase bone marrow cells examined (Figure 2G). Concurrent next generation sequencing looking for gene aberrations associated with myeloid neoplasms detected three different alterations; *ETV6* exon 3 inframe insertion W69dup (allele variant frequency 42%), a *JAK2 R564L* point mutation (allele variant frequency 46%), and a *SF3B1 E622D* point mutation (allele variant frequency 40%). 

#### 3.1.3. Case 3

The patient is a 67-year-old male patient with a history of hypertension, hyperlipidemia, and seizure disorders who presented with severe pancytopenia in the setting of a COVID-19 infection. The LDH was 207 IU/L at presentation. At presentation, an abdominal ultrasound revealed a normal spleen measuring 11.4 cm, however, subsequent follow-up CT scans detected mild splenomegaly measuring from 14.4 to 15.2 cm with multiple splenic infarcts. 

At presentation, the patient had profound pancytopenia with macrocytic anemia. His CBC revealed a Hb of 7.5 g/dL with a MCV 103 fl, a WBC count of 1.0 K/uL, an ANC of 0.14 K/uL, and a platelet count of 14 K/uL. The patient never developed leukocytosis or thrombocytosis during follow up. 

**Figure 3:**
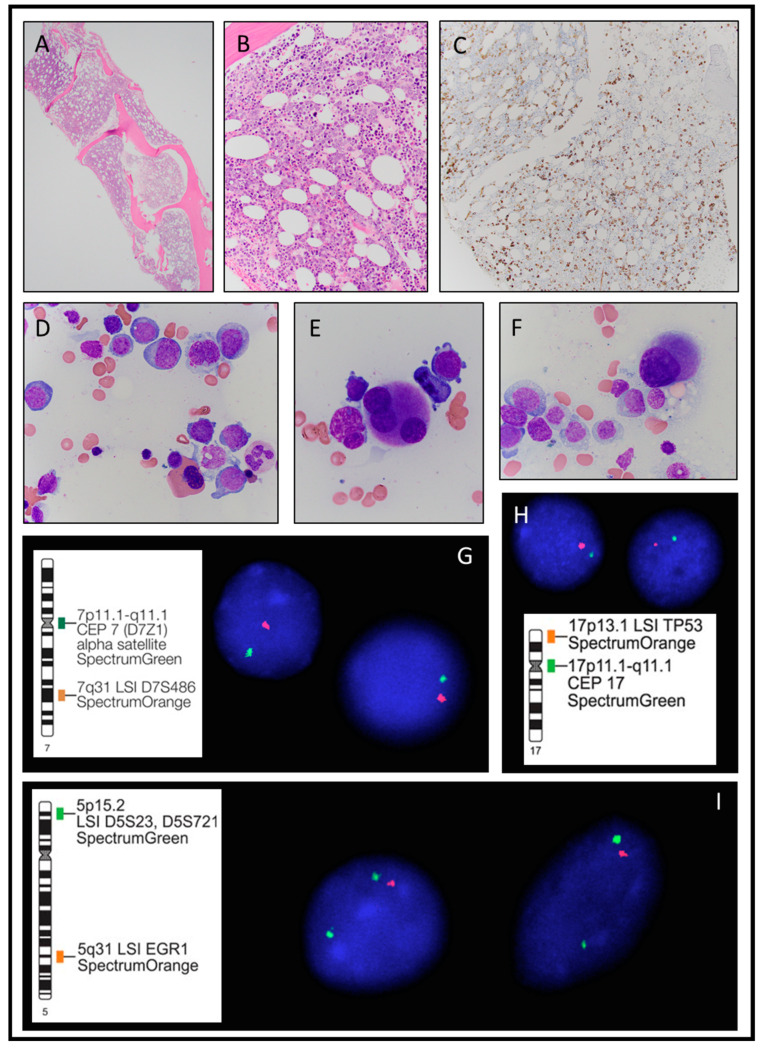
Case 3. (**A**) Bone marrow core biopsy with approximately 70% cellularity (2×). (**B**) Bone marrow core biopsy with trilineage hematopoiesis with left-shifted maturation (40×). (**C**) CD34 highlights excess blasts on core biopsy (10×). (**D**) Bone marrow aspirate with significant dyserythropoiesis and dysgranulopoiesis (100×). (**E**) Dysplastic micromegakaryocyte with small hypolobated nucleus (100×). (**F**) Dysplastic small megakaryocytes with disjointed nuclear lobes (100×). (**G**) Fluorescence in situ hybridization (FISH) analysis with Abbott Molecular D7S522 and CEP7 dual color probe set (Spectrum Orange directly labeled D7S522 probe for the 7q31 region and Spectrum Green directly labeled probe for the centromeric region of chromosome 7) detects monosomy 7. (**H**) Fluorescence in situ hybridization (FISH) analysis with the Abbott Molecular TP53 and CEP17 dual color probe set (Spectrum Orange directly labeled probe for the TP53 gene on 17p13 and Spectrum Green directly labeled probe for the centromeric region of chromosome 17, consistent with loss of the centromere of chromosome 17 and deletion of the TP53 gene. (**I**) Fluorescence in situ hybridization (FISH) analysis with the Abbott Molecular EGR1 dual color probe set (Spectrum Orange directly labeled probe for the EGR1 gene on 5q31 and Spectrum Green directly labeled probe for D5S23/D5S721 on 5p15.2) detects deletion of the 5q31 region.

The circulating blasts varied from 2% to 6% at presentation. A bone marrow biopsy from an outside institution revealed an expanded blast population representing approximately 11% of bone marrow aspirate differential counts and the CD34 on the core biopsy detected 15–19% blasts. A diagnosis of myelodysplastic syndrome with excess blast-2 was entertained initially that subsequently transformed to acute myeloid leukemia at a follow up biopsy. The second follow up biopsy was performed at our institution approximately from two to three weeks later. The bone marrow core biopsy was hypercellular with a cellularity of approximately 60–70% (Figure 3A,B). The bone marrow aspirate revealed significant trilineage dysplasia with dysgranulopoiesis, dyserythropoiesis, and dysmegakaryopoiesis (Figure 3D–F). The blasts represented approximately 22% of the bone marrow aspirate differential counts and CD34 highlighted 20–25% blasts with significant clustering and scattered aberrant megakaryocytes (Figure 3C). The CD42b additionally highlighted increased megakaryocytes with numerous small and hypolobated forms. 

Corresponding flow cytometry studies detected an abnormal population of CD34 positive myeloblasts accounting for approximately 15% of the total analyzed events. The corresponding cytogenetic studies detected a profoundly complex abnormal karyotype with four clones and the FISH studies detected deletion 5q31, monosomy 7, monosomy 17, and deletion of *TP53*. Concurrent targeted next generation sequencing looking for myeloid neoplasm associated gene aberrations detected three different alterations: a *NF1 I679fs* insertion mutation (allele variant frequency 17%), a *JAK2 I670V* point mutation (allele variant frequency 44%), and a *TP53* point mutation (allele variant frequency 58%). 

## 4. Discussion

In this manuscript, we presented three unique cases of myelodysplastic syndrome with diverse *JAK2* mutations. To our knowledge, little is known in the literature about the correlation of *JAK2* mutation variants and the phenotype of myeloid neoplasms, particularly MDS. The first case is an excellent example of the diagnostic challenges that arise when confronted with sequential findings that may lead to a controversial diagnosis. The morphologic features were overall insufficient alone for a specific diagnosis and the consequent cytogenetic and molecular findings that revealed additional information that was relevant for more specific diagnoses. At presentation with progressive normocytic anemia, a hypocellular bone marrow, and pending cytogenetic and molecular findings, the differential diagnosis included aplastic anemia (AA), a hypoplastic MDS with fibrosis, and paroxysmal nocturnal hemoglobinuria (PNH). Flow cytometry excluded a PNH clone. The cytogenetic finding of a deletion of 7q31 at presentation was presumptive of a myeloid neoplasm more likely to be a hypocellular myelodysplastic syndrome with fibrosis that fitted with the morphologic findings according to the current WHO classification. However, surprisingly, the concurrent findings of a *JAK2 V617F* mutation raised more challenging questions and included in the differential diagnosis PMF or a hybrid MDS/MPN neoplasm that was unclassifiable. According to the current WHO cytogenetic criteria, 7q- is presumptive of myelodysplastic syndrome and is associated with an unfavorable prognosis. Interestingly, the inherited bone marrow failure molecular panel detected a heterozygous *SAMD9* variant of indeterminate significance. The pathogenic variants in *SAMD9* have been associated with bone marrow failure and a predisposition for monosomy 7 myelodysplasia [25,26,27]. A family study of affected and unaffected individuals may help shed further light on the significance of such findings in our patient. It may be safe in cases with such discordant and challenging findings to report this myeloid neoplasm or presumptive myelodysplastic syndrome with myelofibrosis as unclassifiable until the full phenotype reveals itself. Especially, in this patient, a JAK2 V617F mutation with a low allele variant frequency was detected, with mutations usually associated with myeloproliferative neoplasms. The low allele variant frequency may be explained by a low level of disease burden at this stage of the disease that has not fully expressed itself. It should be noted that myelofibrosis in MDS is an independent poor prognosticator (3). In addition, the patient smoking history raised further questions about potential causative associations with *JAK2* mutations as erythrocytosis in smokers was suggested to render cells more susceptible for such mutations [28]. The patient’s calculated revised IPSS score was an intermediate risk with a total of 4 points (3 points for 7q- and 1 point for an Hb 8–10 g/dL). Due to the patient’s predicted prognosis, he is under consideration for a stem cell transplant.

The second unique case is another example of a challenging situation where correlation with clinical morphologic features, cytogenetic findings, and molecular findings raises questions on the role of *JAK2* mutations on the myeloid neoplasm phenotype. The patient presented with progressive macrocytic anemia without evidence of leukocytosis or thrombocytosis. The cytogenetic findings were non-specific. While the molecular findings with a *JAK2 R564L* point mutation suggested an overlap MDS/MPN syndrome, the presence of markedly increased ring sideroblasts in the absence of thrombocytosis argued against the diagnosis of an MDS/MPN-RS-T. MDS/MPN-RS-T is a distinct MDS/MPN overlap syndrome entity that is usually associated with both *SF3B1* and *JAK2 V617F* mutations. In this entity, it was found that the *SF3B1* mutation drives the formation of ring sideroblasts and that the *JAK2 V617F* mutation drives the thrombocytosis. In our case, due to the absence of leukocytosis and thrombocytosis or significant evidence of a myeloproliferative neoplasm, the patient was considered most in keeping with a myelodysplastic syndrome with multilineage dysplasia and ring sideroblasts. The patient’s calculated revised IPSS score was intermediate risk with a total of 3.5 points (1.5 points for a karyotype with 1q and 2 points for a Hb less than 8 g/dL). The patient became transfusion dependent during the follow up and was treated with azacitidine and venetoclax with a subsequent plan for allogenic bone marrow transplantation.

This case raises the discussion about the potential impact of different *JAK2* mutations and downstream transcription regulators on the clinical and morphologic phenotype of myeloid neoplasms. Interestingly, during our search for cases with *JAK2* mutation variants, we came across a case with features of a myeloproliferative neoplasm with thrombocytosis and a *JAK2 R564Q* (allele variant frequency 43%) point mutation. In addition, *JAK2* exon 12 in frame deletion mutations, spanning from residues 536 to 547 have also been reported in MPN particularly in patients with PV who were negative for the common *JAK2 V617F* mutation [7,8]. However, the *JAK2 R564L* variant has not been well described in the literature and its clinical significance is unclear.

The third unique case presented with high grade morphologic features with excess blasts and evolution to acute myeloid leukemia. The cytogenetic findings revealed a markedly complex karyotype with cytogenetic aberrations commonly found in MDS including deletion 5q31 and monosomy 7. This patient also revealed a different *JAK2* variant mutation, *JAK2 I670V*, which is not in exon 12 and has not been well described in the literature. However, the high allele variant frequency burden suggests this mutation to be an early event. In addition, the patient’s molecular studies revealed a *TP53* point mutation that is associated with an aggressive clinical course. The patient was initially treated with Vyxeos (daunorubicin–cytarabine) and then the treatment changed to Decitabine and venetoclax. The patient is currently hospitalized with recovering absolute neutropenia, persistent anemia, and thrombocytopenia. This patient has high risk features of relapse based on morphology, cytogenetic, and molecular findings. 

## 5. Conclusions

In summary, our manuscript reached three main conclusions. First, *JAK2 V617F* mutations are extremely rare in MDS and, in its presence, an MPN must be excluded. Second, *JAK2* mutations are diverse and JAK2 variant mutations may lead to a myelodysplastic syndrome phenotype. Third, *JAK2* variants such as *JAK2 R564L* and *JAK2 I670V* may be seen in association with a myelodysplastic phenotype; however, their significance in MDS pathogenesis is uncertain. While the *JAK2 I670V* mutation has been reported at low frequency in the European population (https://gnomad.broadinstitute.org/ accessed on 3 October 2022), its exact impact on the pathogenesis of MDS may be arguable and further studies are recommended to investigate the relationship between *JAK2* mutation variants and their clinico-morphologic phenotypes. The number of cases with *JAK2* mutations were small in this study and had variable prognosis with transformation to acute myeloid leukemia in a case, hence in our opinion committing to any definitive conclusion about the prognostic relevance of such mutations and providing definitive management guidelines would be challenging and needs more multi-institutional collaborative studies on a larger sample of cases.

## Figures and Tables

**Table 1:** JAK2 mutation in the Literature.

Literature (Reference Number)	Population of Concern to Our Study	Number of Participants in Study	*JAK2* Mutation/Other Studies	Frequency (Number of Cases) with JAK2 V617F Mutation/Variants	Conclusions
Nielson C. et al. [1]	General healthy	10,507	*JAK2 V617F*	0.2% (18)	JAK2 V617F is very rare in the general healthy population and is associated with increased morbidity and mortality.
Nielson C. et al. [4]	General healthy	49,488	*JAK2 V617F*	0.1% (68)	*JAK2 V617F* somatic mutation in general healthy participants has a high diagnostic value for myeloproliferative neoplasms when combined with hematological indices.
Trelinski J. et al. [5]	E.T.	43	*JAK2 V617F*		Impaired apoptosis of megakaryocytes and bone marrow mononuclear cells.
Schulze et al. [9]	MF (PMF, post-ET-MF, and post-PV-MF)	128	*JAK2 V617F* *MPL*	14.6% (82) JAK2R1063H (6) JAK2R893T(1) JAK2T525A(1) MPLY591D(3) MPLW515 L(2) MPLE335K(1)	Recurrent concomitant classical and/or noncanonical *JAK2*- and *MPL*-mutations detected in 15.7% of *JAK2V617F*- and *MPLW515*-positive MF patients and appear to express genotype—phenotype associations.
Fermo et al. [10]	MDS	53	*JAK2 V617F*	5% (3)	In MDS, *JAK2 V617F* has a low prevalence and identifies a subset with proliferative characteristics.
Bejar et al. [12]	MDS	439	*JAK2*	3% (13)	*JAK2* mutations are rare in MDS.
Haferlach et al. [13]	MDS	944	*JAK2*	4–5%	RARS-T usually associated with *JAK2* and *SF3B1* co-mutations with lower *JAK2* mutation burden suggesting they evolved from RARS or RCMD-RS.
Sangiorgio et al. [14]	MDS with del(5q)	47	*JAK2 V617F*	12.7% (6)	*JAK2*-mutated myeloid neoplasms with isolated del(5q) show overlap MPN/MDS features
Ingram et al. [15]	MDS with del(5q)	97	*JAK2 V617F*	6.2% (6)	JAK2-mutated cases with deletion 5q are usually hypercellular. It is unclear whether the JAK2 mutation is an early or late event.
Patnaik et al. [17]	MDS with del(5q)	88	*JAK2 V617F*	6.4% (5)	No significant difference in blood counts or clinical outcome between patients with and without *JAK2 V617F*.
Ohyashiki et al. [18].	MDS with and without fibrosis	38 (MDS without fibrosis) +6 (MDS with fibrosis)	*JAK2 V617F*	(2)	MDS with fibrosis may sometimes be associated with *JAK2 V617F*.
Wan Z. et al. [19]	MDS	3100	*JAK2*	2.88%	Meta-analysis with extensive literature review. *JAK2* mutations are rare in MDS.
Steensma et al. [20]	MDS	101	*JAK2 V617F*	5% (5)	*JAK2 V617F* mutation is infrequent in MDS.
Olsen R et al. [21]	MPN/MDS and MDS with fibrosis	45	*JAK2 V617F*	0% in non-MPN cases	*JAK2 V617F* is useful in discriminating MDS with fibrosis from MPN cases.
S.F Yip et al. [22]	MDS with and without fibrosis	186 include 39 assessed for JAK2	*JAK2 V617F*	0%	*JAK2 V617F* is unlikely to play a role in MDS with or without fibrosis biology and MPNs need to be strictly excluded.
Benoit de Renzis et al. [23].	MDS	132	*JAK2 V617F*	(37).	*JAK2 V617F* is associated with a lower incidence of progression to AML and better overall survival.
Lindsley R.C. et al. [24].	MDS before and after stem cell transplant	1514	*JAK2 V617F*	2% (28)	*JAK2 V617F* mutation was associated with shorter survival and higher rate of death without relapse after transplant. High-intensity conditioning regimens may not benefit patients with *JAK2* mutations. May benefit from *JAK2* inhibitors.

**Table 2:** Cases clinical and pathological findings.

C	G	Clinical Presentation	Hb g/dL	MCV fl	WBC K/µL	ANC K/µL	Plt K/µL	LDH IU/L	Pathologic Diagnosis at Presentation	Karyotype FISH	*JAK2* Mutation Variant(VAF)
1	M	Progressive anemia	9.5	88	5.0	3.04	293	164	Hypoplastic MDS with fibrosis	46,XY,+1,der(1;7)(q10;p10)[10]/46,XY[10] FISH: Deletion 7q31	*JAK2 V617F* (8%)
2	F	Progressive macrocytic anemia	8.4	111	5.7	2.90	162	178	MDS-RS-MLD	46,XX,dup(1)(q21q32)[11]/46,XX[9] FISH: Gain of the long arm of chromosome 1	*JAK2 R564L* (46%)
3	M	Pancytopenia	7.5	103	1.0	0.14	14	207	MDS-EB2	Complex karyotype with four clones 45,XY,der(4)t(4;17)(q21;q11.2),add(5)(q11.2),-7,-17,+r[1]/43,XY,der(4)t(4;17)(q21;q11.2),add(5)(q11.2),-7,dic(7;12)(q32;q15),-17[8]/43,XY,der(4)t(4;17)(q21;q11.2),add(5)(q11.2),-7,dic(7;12)(q32;q15),-17, add(21)(p11.2)[6]/ 42,XY,der(4)t(4;17)(q21;q11.2),add(5)(q11.2),-7,dic(7;12)(q32;q15),-17,-21, add(22)(q11.2)[4]/46,XY[1] FISH: Deletion 5q31, monosomy 7, monosomy 17 with deletion of *TP53*.	*JAK2 I670V* (44%)
C: Case number, G: Gender, M: Male, F: Female, Hb: Hemoglobin, MCV: Mean Corpuscular Volume, WBC: White Blood Cell, ANC: Absolute Neutrophil Count, Plt: Platelet, LDH: lactate dehydrogenase											

## Data Availability

Not applicable.
